# A Dual Sensor for pH and Hydrogen Peroxide Using Polymer-Coated Optical Fibre Tips

**DOI:** 10.3390/s151229893

**Published:** 2015-12-17

**Authors:** Malcolm S. Purdey, Jeremy G. Thompson, Tanya M. Monro, Andrew D. Abell, Erik P. Schartner

**Affiliations:** 1 ARC Centre of Excellence for Nanoscale BioPhotonics, Adelaide 5005, SA, Australia; jeremy.thompson@adelaide.edu.au (J.G.T.); tanya.monro@unisa.edu.au (T.M.M.); andrew.abell@adelaide.edu.au (A.D.A.); erik.schartner@adelaide.edu.au (E.P.S.); 2 Institute for Photonics and Advanced Sensing (IPAS), The University of Adelaide, North Terrace, Adelaide 5005, SA, Australia; 3 Discipline of Chemistry, School of Physical Sciences, The University of Adelaide, North Terrace, Adelaide 5005, SA, Australia; 4 Robinson Research Institute, School of Medicine, The University of Adelaide, North Terrace, Adelaide 5005, SA, Australia; 5 University of South Australia, North Terrace, Adelaide 5001, SA, Australia

**Keywords:** optical fibre, hydrogen peroxide probe, pH sensor, dual sensor, fibre tip sensor

## Abstract

This paper demonstrates the first single optical fibre tip probe for concurrent detection of both hydrogen peroxide (H_2_O_2_) concentration and pH of a solution. The sensor is constructed by embedding two fluorophores: carboxyperoxyfluor-1 (CPF1) and seminaphtharhodafluor-2 (SNARF2) within a polymer matrix located on the tip of the optical fibre. The functionalised fibre probe reproducibly measures pH, and is able to accurately detect H_2_O_2_ over a biologically relevant concentration range. This sensor offers potential for non-invasive detection of pH and H_2_O_2_ in biological environments using a single optical fibre.

## 1. Introduction

Hydrogen peroxide (H_2_O_2_) and pH play vital combined roles in cellular signalling [1,2,3], tumour development [4,5,6,7] and reproductive health science [8,9,10,11]. For example, the unregulated production of H_2_O_2_ by an embryo is a hallmark of embryonic stress [12], while pH fluctuations during embryo culture can negatively affect embryonic development [13]. The simultaneous detection of pH and H_2_O_2_ would therefore provide significant benefit in monitoring the associated cellular processes. H_2_O_2_ and pH can be detected in cells by specific fluorophores, measuring either an increase in fluorescence intensity [14,15], or a change in emission spectra respectively [16,17]. However the use of these fluorescent probes in applications such as *in vitro* fertilisation (IVF) poses significant scientific and ethical questions, as their effect on the development of embryos is unknown. As such, direct contact of fluorophores with an embryo is ethically unsound and not allowable in most regulatory jurisdictions. 

Optical fibre-based probes offer an attractive and non-invasive approach. Here a fluorophore of interest can be attached to the fibre surface for localised measurement without being released into the solution [18,19,20]. Various configurations of optical fibres have been examined for development of such fluorescent sensors, specifically; functionalized end-faces (tip sensors) [21,22], exposed core [23] and microstructured fibres [24,25]. Although microstructured fibre based sensors can be more sensitive than tip sensors [26], filling of the air holes with analyte is required in order to perform a measurement. This typically restricts microstructured fibres to single temporal measurements, unless microfluidics or external flushing systems are employed, and these may impede on the cell culture environment. Exposed-core fibre sensors are ideal for environmental sensing and do not require microfluidics or external flushing and offer advantages in distributed sensing. However, tip-based sensors offer potential for temporal measurements in a single location rather than distributed along the length, or can be repositioned to obtain a spatial map of the sample as desired. Tip sensors often have reduced sensitivity compared to microstructured fibres [26], especially as conventional attachment of a single layer of fluorophore to a fibre tip results in a low signal intensity [27]. However, the signal intensity can be improved by increasing the density of fluorophore on the fibre tip. 

**Figure 1:**
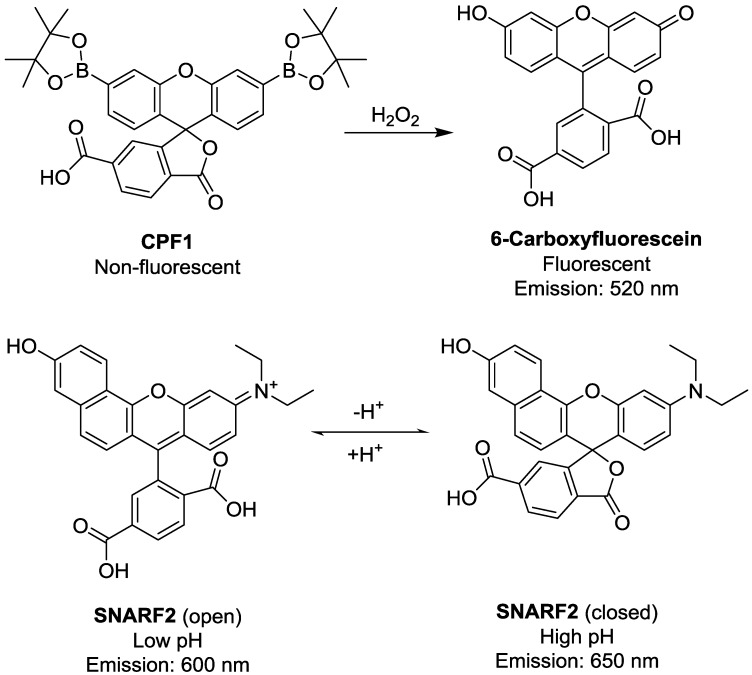
Chemical structures of fluorescent probes used in this study. Carboxyperoxyfluor-1 (CPF1) reacts with H_2_O_2_ to form the fluorescent 6-carboxyfluorescein. Seminaphthorhodofluor-2 (SNARF2) is found in the protonated (open) form and lactone (closed) at low and high pH respectively.

H_2_O_2_ can be detected by aryl boronate-based fluorophores such as peroxyfluor-1 (PF1) [28] and carboxyPF1 (CPF1, Figure 1) [29]. These aryl boronates have been shown as particularly effective fluorescent probes for detection of H_2_O_2_ in human spermatozoa and bovine oocytes [29,30]. pH can be detected using a range of fluorophores, with seminaphthorhodofluor-2 (SNARF2, Figure 1) offering some advantages over alternative probes, as the ratiometric emission from this probes changes its spectral features over the physiological pH range, with a pK_a_ of 7.5 [31]. This minimises potential errors which could arise from using a solely intensity-based probe. Additionally, its emission spectrum overlaps minimally with the emission of CPF1 [32], allowing the separate interrogation of each fluorophore. 

This paper reports the first dual probe for sensing pH and the detection of H_2_O_2_ by immobilising two separate fluorophores (CPF1 and SNARF-2) onto a single optical fibre tip in a polyacrylamide matrix. The two fluorophores are attached to a multi-mode fibre tip by a light-catalysed polymer coating [33], to allow for greater control of fluorophore surface density and thus subsequent signal intensity. This then allows detection of both H_2_O_2_ and pH within a single system.

## 2. Experimental Section 

### 2.1. Materials

All chemicals were purchased from Sigma-Aldrich unless otherwise stated. Bis(acrylamide) was purchased from Polysciences (Warrington, PA, USA). HPLC grade acetonitrile was purchased from Scharlau. 100 mM Phosphate buffer solutions were prepared from monosodium phosphate and disodium phosphate in Milli-Q water. Multimode fibre (200 µm core diameter, FG200UCC) was purchased from Thorlabs (Newton, NJ, USA), with one end connectorised for attachment to the optical setup.

### 2.2. Polyacrylamide Photo-Polymerisation on Optical Fibre Tips

A solution of 3-(trimethoxysilyl)propyl methacrylate (5 μL) in 10% acetic acid solution (30 μL) and ethanol (1 mL) was mixed and sonicated until clear. Multi-mode fibre was cleaved and each segment immersed in the methacrylate solution for 1 h. The fibre tip was then dried under N_2_, rinsed with Milli-Q water and re-dried under N_2_. The distal end of the fibre was coupled into a 405 nm source (Crystalaser 405 nm) using a 10× microscope objective. A monomeric stock solution comprising of 3% bisacrylamide, 27% acrylamide and 70% pH 6.5 phosphate buffer solution was dissolved under sonication. CPF1-NHS (0.2 mg), and SNARF2-NHS (0.2 mg) were added to this solution (400 µL) and 200 µL of the resulting solution was pipetted into a small Eppendorf tube. Triethylamine (10 µL/mL) was added to the mixture, and the fibre tip was immersed in this solution exactly 60 s after addition of the triethylamine and immediately irradiated under 405 nm light for 10 s at 13.4 mW, to form a polymeric coating on the fibre tip. 

### 2.3. Optical Measurements

**Figure 2:**
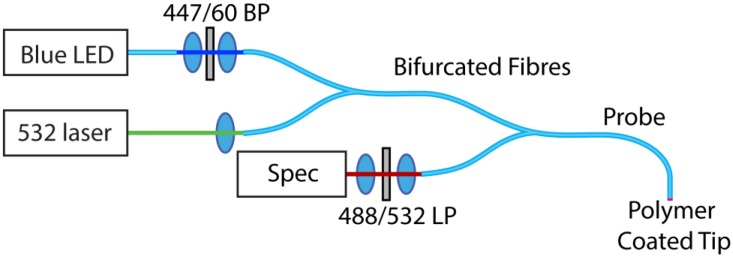
Experimental configuration for optical measurements of the combined pH/peroxide sensor. The blue LED source was used to illuminate the peroxide sensitive carboxyperoxyfluor-1 (CPF1) fluorophore, while the green excites the pH sensitive seminaphtharhodafluor-2 (SNARF).

The experimental configuration used for optical measurements is shown in Figure 2 below.

A 470 nm blue LED source (Thorlabs M470F1, Newton, NJ, USA) with an appropriate bandpass filter (Semrock Brightline 447/60, Rochester, NY, USA) was coupled into one input of a bifurcated fibre (Ocean Optics 200 µm, UV/VIS). Attached CPF1 was then excited with light from a 532 nm green laser (Crystalaser 25 mW, Reno, NV, USA) coupled into the other input for excitation of the SNARF. An additional bifurcated fibre was used to connect the excitation sources to the sensing fibre, with the remaining input connected to the input of the spectrometer (Horiba iHR550, Synapse detector, Kyoto, Japan). Long-pass filters were inserted directly into the spectrometer input cage, with 488 nm (Semrock 488 nm Edgebasic) and 532 nm (Semrock 532 nm Razoredge) used for peroxide and pH respectively.

The two excitation channels were controlled independently, with only one excitation wavelength used at any particular time to excite either the peroxide or pH channel. The corresponding emission filter was used with each excitation source to attenuate residual pump light from the fibres. The use of connectorised fibres and multi-mode fibres greatly simplifies the measurement procedure, as no adjustments or realignment are required when swapping between pH and peroxide measurements.

## 3. Results and Discussion

### 3.1. Hydrogen Peroxide Detection 

#### 3.1.1. Detection of Biologically Relevant H_2_O_2_ Concentrations

**Figure 3:**
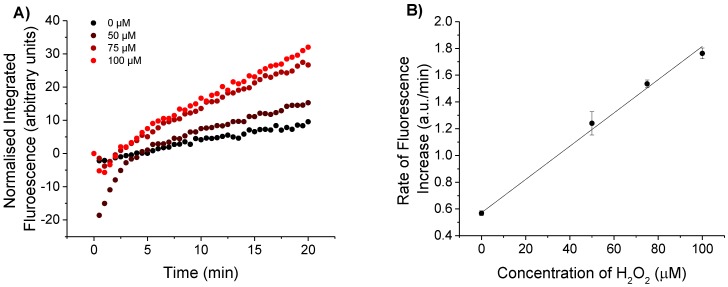
(**A**) Integrated fluorescence intensity from CPF1 using blue excitation with varied peroxide concentration in pH 7.5 buffer; 100 μM H_2_O_2_ shows an increased fluorescent response from the fibre without H_2_O_2_; (**B**) Slope of integrated fluorescence for increasing concentrations of H_2_O_2_ (0, 50, 75 and 100 μM). Error bars indicate the standard error of the calculated slope.

CPF1 and SNARF2 immobilised on fibre tips were tested in solutions containing H_2_O_2_ to establish the sensitivity of this surface configuration. Fibre tips were first functionalised with 3-(trimethoxysilyl)propyl methacrylate, then dipped into a solution of acrylamide/bisacrylamide with N-hydroxysuccinimide esters of CPF1 and SNARF2. The N-succinimide esters of CPF1 and SNARF2 increase solubility in the acrylamide solution to provide a more reproducible density of fluorophores embedded in the polymer matrix. Excitation light (405 nm) was coupled into the distal end of the fibre and the tip irradiated for 10 s to form a polymer layer on the tip containing the fluorophores. The functionalised fibres were dipped into a range of concentrations of H_2_O_2_ (0, 50, 75 and 100 μM) in pH 7.5 phosphate buffer, and the emission peaks from CPF1 at 520 nm and SNARF at 600 and 660 nm were observed under 473 nm excitation. A low excitation power was used (27 μW) for these trials to minimise any potential effects of photobleaching. The entire spectrum was then integrated and normalised to the initial peak of CPF1 at 520 nm. This was necessary because each probe has slightly different initial fluorescence values and hence raw intensity values cannot be directly compared. Furthermore, each fibre probe was only used once for detection of H_2_O_2_ to ensure maximum consistency between trials. Figure 3 shows an increase in normalised integrated fluorescence due to CPF1 over a 20 min exposure to H_2_O_2_. This time interval was dictated by the reaction rate of aryl boronates such as CPF1 with H_2_O_2_ [34].

Normalised fluorescence of CPF1 in the presence of 100 μM H_2_O_2_ is greater than for the control, which lacked H_2_O_2_ (Figure 3A). This increase in fluorescence is consistent with CPF1 reacting with H_2_O_2_ on the fibre tip. Furthermore, a plot of the rate of increase in fluorescence vs concentration of H_2_O_2_ (Figure 3B) clearly shows this rate increasing as the concentration of H_2_O_2_ increases from 0 μM to 50 μM, 75 μM and 100 μM of H_2_O_2_. A similar increase was observed in our previous studies on the detection of H_2_O_2_ with CPF1 in solution [27], and we have also shown that CPF1 is able to detect relevant H_2_O_2_ concentrations in reproductive biology [29]. Hence, this probe exhibits sufficient sensitivity for this biological environment.

An initial drop in fluorescence was observed for some probes (see Figure 3A), particularly the probe immersed in a 50 μM H_2_O_2_ solution. This is likely due to a change in emission properties of the fluorophores as the probe is moved from air into the solution. A more rapid increase in fluorescence was observed in the first 5 min, suggesting the probe is equilibrating in the new medium. After 5 min the probes show a near linear increase in fluorescence intensity. Thus, an incubation time of greater than 5 min is required to give an accurate indication of the rate of increase in fluorescence due to H_2_O_2_. A plateau was not observed over the time course of the experiment, suggesting quantitative data should be obtained from the rate of increase in fluorescence rather than the overall increase in fluorescence.

It is also important to note that an increase in the integrated fluorescent signal was evident, even in the absence of H_2_O_2_ (Figure 3A). A decrease in fluorescence would be expected if photobleaching or leaching of the fluorophore from the polymer occurred over the course of the experiment. The effect of photobleaching on CPF1 was of particular interest, since CPF1 is oxidised by H_2_O_2_ to give 5-carboxyfluorescein. 5-carboxyfluorescein is known to photobleach [35], a process which occurs more rapidly in the presence of reactive oxygen species such as H_2_O_2_ [36]. In order to accurately sense H_2_O_2_ with CPF1, low rates of photobleaching must be achieved. An increase in fluorescence in the absence of H_2_O_2_ suggests that photobleaching was not occurring on the fibre during the experiment. This is an important observation for the practical use of the probe since the fibre’s ability to sense accurately would be reduced by photobleaching of the fluorophores on the fibre tip.

#### 3.1.2. Effect of Change in pH on Detection of H_2_O_2_

**Figure 4:**
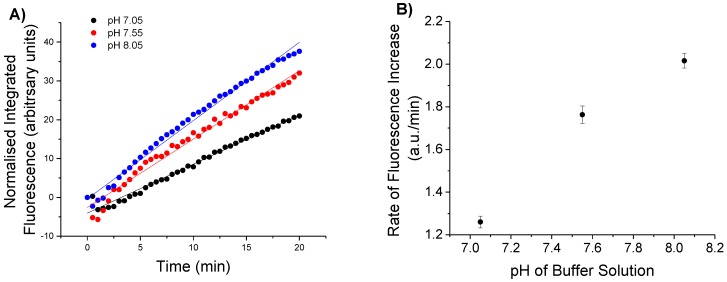
Response of CPF1 to 100 μM H_2_O_2_ in solutions that varied in pH. (**A**) Integrated fluorescent responses of probes to 100 μM H_2_O_2_ in pH 7.05, 7.55 and 8.05 over 20 min using blue excitation; (**B**) Rate of increase in fluorescence of each probe with H_2_O_2_ in each of the 3 pH solutions. Error bars indicate the standard error of the calculated slope.

The effect of pH on the detection of H_2_O_2_ in fibre was next investigated by immersing functionalised fibre tips in solutions of H_2_O_2_ of differing pH. Fibre tips were functionalised with CPF1 and SNARF2 as before and separately immersed in 100 μM separate solutions of H_2_O_2_ at a pH of 7.05, 7.55 or 8.05. A 470 nm light source was coupled into the fibre for excitation and the increase in fluorescence was recorded over 20 min. All spectra were integrated and normalised as before, with the results shown in Figure 4A. The rate of increase in fluorescence was calculated as depicted in Figure 4B. The observed rate at pH 8.05 was 1.5 times greater than the rate observed at pH 7.05 (Figure 4B). 

The reaction rate of aryl boronates (such as CPF1) with H_2_O_2_ is higher in more basic solutions [37]. CPF1 reacts with the conjugate base of H_2_O_2_ (hydroperoxide ion HOO^−^) [15] to give fluorescent 5-carboxyfluorescein. As the pH increases, more H_2_O_2_ dissociates into its conjugate base, HOO^−^. The concentration of HOO^−^ available to react with CPF1 will therefore be higher in more basic solutions, accounting for the observed upward trend in rates due to increasing pH (Figure 4B). Furthermore, the product of CPF1 with H_2_O_2_ is a carboxyfluorescein, and fluorescein exhibits different quantum yields of fluorescence at differing pH [38]. Thus, the pH of the solution is highly pertinent to the accurate detection of H_2_O_2_. This is further highlighted by a comparison of rates of increase in fluorescence at different pH and H_2_O_2_ concentration, shown in Figure 3B and Figure 4B. A rate of approximately 1.2 a.u./min was calculated for a 100 μM solution of H_2_O_2_ in pH 7.05 (Figure 4B). However, a similar rate was calculated for a 50 μM solution of H_2_O_2_ at a pH of 7.55 (Figure 3B). The observed rate in these experiments is clearly dependent on the pH, in addition to the concentration of H_2_O_2_. It is hence necessary that the pH of a solution must be known in order to calculate an unknown concentration of H_2_O_2_ resulting from an increase in fluorescence. Therefore, it is critical that the probe also incorporates a pH sensitive component to accurately determine the H_2_O_2_ concentration. This improves upon many systems for detection of H_2_O_2_ that do not simultaneously measure pH, despite the reported effect on H_2_O_2_ detection [15].

### 3.2. pH Sensing

#### 3.2.1. Initial pH Sensing

**Figure 5:**
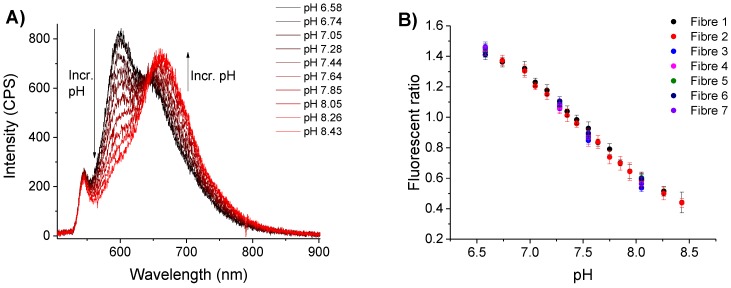
pH response of SNARF-2 embedded in polyacrylamide on fibre tip to varied pH. (**A**) Emission spectra of SNARF in various pH buffers; (**B**) Ratio of emission peak intensities 600/660nm shown with over multiple trials. The effect of noise was reduced by taking the mean of eight values between 598–602 nm and 558–662 nm. Error bars represent the standard deviation of these values.

The sensitivity of these functionalised fibre tips was defined across a series of solutions of differing pH, ranging from 6.5 to 8.5. Fibres functionalised with CPF1 and SNARF2 as before were dipped into phosphate buffer solutions of each pH. 532 nm light attenuated to 13 μW was coupled into the fibre for excitation, and the fluorescent signal from immobilised SNARF2 was collected after 1 min equilibration time. The fibre was removed from solution, dried, and immersed in a subsequent buffer solution. Two fibre probes were calibrated in this way using sixteen buffer solutions ranging from pH 6.5 to 8.5 as shown in Figure 5. This broad pH range (6.4–8.5) was chosen in order to demonstrate the potential of the probe in biological applications beyond the narrower constraints of an embryo. Five additional probes were then calibrated in selected solutions across this range (see fibres 3–7 in Figure 5B). The fluorescence spectra were recorded as shown in Figure 5A. The probe exhibits a decrease in intensity of fluorescence at 600 nm as the pH increases, with an increase in intensity at 660 nm.

The pH of the buffer was correlated with the observed fluorescent signal by calculating the ratio of intensities at 600 nm and 660 nm for each spectrum (Figure 5B). This analysis revealed an inverse correlation between this fluorescent ratio and pH of the solution. The plot also shows a linear trend over the pH range 7.0 to 8.0 and indicates that the sensor should be of use for determining the pH of a biological sample near physiological pH. Moreover, multiple fibre trials produced similar pH calibration curves (Figure 5B) showing good reproducibility between fibres. This data also indicates that SNARF2 bound to a fibre tip behaves as in solution [32]. Therefore, SNARF2 effectively senses the pH of a buffer solution when embedded in polyacrylamide on a fibre tip.

#### 3.2.2. pH Sensing before and after Detection of Hydrogen Peroxide

**Figure 6:**
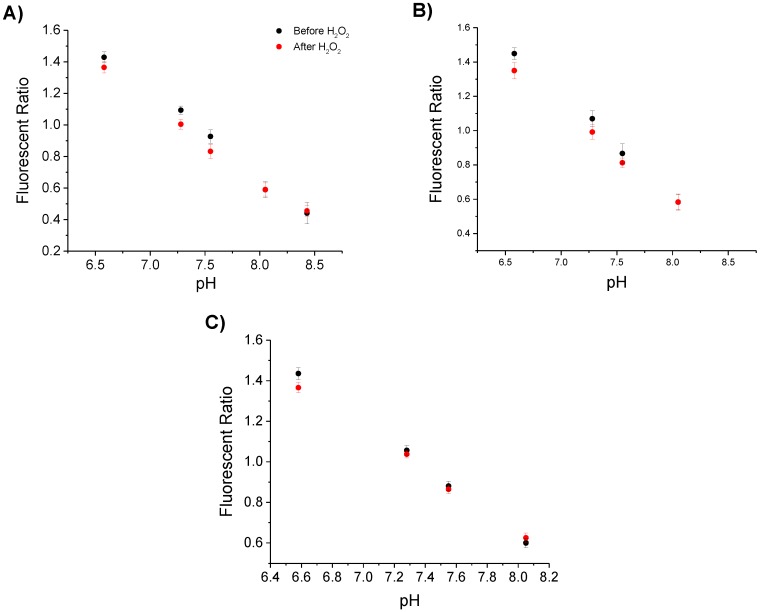
Sensing of pH before and after immersion in H_2_O_2_. Each graph plots the ratio of emission peaks of SNARF2 at 600/660 nm with the pH of the buffer solution tested, before and after solutions: (**A**) 100 μM solution of H_2_O_2_ in pH 7.55 buffer; (**B**) 50 μM H_2_O_2_ in pH 7.55 buffer; (**C**) 100 μM H_2_O_2_ in pH 7.05 buffer. Three different samples were trialled before and after H_2_O_2_ solutions to demonstrate the independence of the result to concentration of H_2_O_2_ or the pH of the H_2_O_2_ solution. To reduce any effect of noise, the mean of eight values between 598–602 nm and 558–662 nm is given. Error bars represent the standard deviation of these values.

The functionalised fibre probes were used to sense pH before and after immersion in H_2_O_2_, in order to determine if this affected the pH sensing capability. Each fibre was functionalised with CPF1 and SNARF2 and calibrated in phosphate buffer solutions of known pH as described in Section 3.2.1. The fibre tips were then immersed for 20 min in one of three H_2_O_2_ solutions: 100 μM solution of H_2_O_2_ in pH 7.55 buffer, 50 μM H_2_O_2_ in pH 7.55 buffer or 100 μM H_2_O_2_ in pH 7.05 buffer. These conditions represent the range of conditions that the probe may experience in unknown samples. Each probe was calibrated again in phosphate buffer solutions as above. The resulting pH calibration curves were plotted for each probe both before and after immersion in H_2_O_2_ (Figure 6). 

The fluorescent ratios shown in Figure 6A show minimal changes before and after immersion in 100 μM H_2_O_2_ in pH 7.55. This indicates that reaction of H_2_O_2_ with CPF1 does not affect the sensing of pH by SNARF2. Figure 6B reveals similar fluorescent ratios before and after immersion in 50 μM H_2_O_2_ in pH 7.55 buffer. H_2_O_2_ again does not affect the sensing of pH over the tested concentration range, 50 μM to 100 μM H_2_O_2_. Figure 6C shows the fluorescent ratios of SNARF on fibre tips before and after immersing in 100 μM H_2_O_2_ in pH 7.05 buffer. As per the previous results, the fluorescent ratio curves did not change significantly after immersion in H_2_O_2_. Importantly, immersing the probe into a solution containing H_2_O_2_ does not affect the pH sensing capability of the probe. This demonstrates that SNARF2 can be used on optical fibre tips for sensing pH independent of the detection of H_2_O_2_ by CPF1.

## 4. Conclusions/Outlook

The tip of an optical fibre has been functionalised with two separate fluorophores, CPF1 and SNARF2 embedded in polyacrylamide, in order to allow measurement of the H_2_O_2_ concentration and pH respectively. The probe is demonstrated to effectively detect H_2_O_2_ over a physiological pH range. The probe shows a minimum detectable concentration of 50 μM H_2_O_2_ at pH 7.5, and pH was measured repeatedly over the range 6.5–8.5 with resolution of 0.1 pH units. Each fluorophore was used in tandem by alternating excitation sources, *i.e.*, blue excitation to interrogate CPF1 for H_2_O_2_ detection, and green excitation for SNARF2 to sense pH, with minimal cross-talk. The combination of pH and H_2_O_2_ detection also addressed the crucial issue of accurate measurement of H_2_O_2_ in solutions with varying or unknown pH, where the pH of the solution alters the apparent H_2_O_2_ concentration. This is the first example of a dual pH and H_2_O_2_ probe and is an important proof of concept for the detection of pH and H_2_O_2_ in ethically complex biological environments such as found in an IVF laboratory.

This probe could find potential application if placed near the cumulus cells of an oocyte for monitoring of extracellular pH and H_2_O_2_ fluxes during fertilisation and during early embryonic development. Tapering of the fibre tip could also increase the resolution from 200 μm to a few microns if required [39]. This fibre probe may offer potential not only in embryology, but a range of biological applications whereby the system must remain isolated from any external agents such as organic fluorophores. 

## Supplementary Files

Supplementary File 1
