# Supplementary material for: A Dual Sensor for pH and Hydrogen Peroxide Using Polymer-Coated Optical Fibre Tips
Source: Sensors (Basel). 2015 Dec 17;15(12):31904–13. doi: 10.3390/s151229893 (PMC4721812; doi:10.3390/s151229893)
Supplement: Supplementary File 1 [file sensors-15-29893-s001.pdf]

Supplementary Materials: A Dual Sensor for pH and Hydrogen Peroxide Using Polymer-Coated Optical Fibre Tips

Malcolm S. Purdey, Jeremy G. Thompson, Tanya M. Monro, Andrew D. Abell and Erik P. Schartner

1. Synthesis of CPF1 and SNARF2

CPF1 N-hydroxysuccinimide ester (CPF1-NHS) (Figure S1) was prepared using a modification of a literature procedure [1]: CPF1 [2] (276 mg, 0.46 mmol), EDC·HCl (157 mg, 0.77 mmol) and N-hydroxysuccinimide (67 mg, 0.5 mmol) were dissolved in anhydrous dimethylformamide (7 mL) under nitrogen and stirred for 1 h. The reaction mixture was poured into diethyl ether (100 mL) and washed with water (100 mL). The aqueous phase was washed with diethyl ether (2 × 100 mL), the organic layers combined and dried over MgSO4. The resultant solution was filtered, evaporated onto celite and eluted through a silica column using 1% methanol in dichloromethane to afford an off-white solid CPF1-NHS (165 mg, 53%) The product was then characterised by 500 MHz 1HNMR in CDCl3 for comparison to literature [1]: δ(ppm) 8.32 (d, 1H, J = 8.1 Hz), 8.15 (dd, 1H, J1 = 8.0 Hz, J2 = 1 Hz), 7.78 (s, 1H), 7.74 (s, 2H), 7.45 (d, 2H, J = 7.8 Hz), 6.81 (dd, 2H, J1 = 7.8 Hz), 2.82 (br. s, 4H), 1.32 (s, 24H).

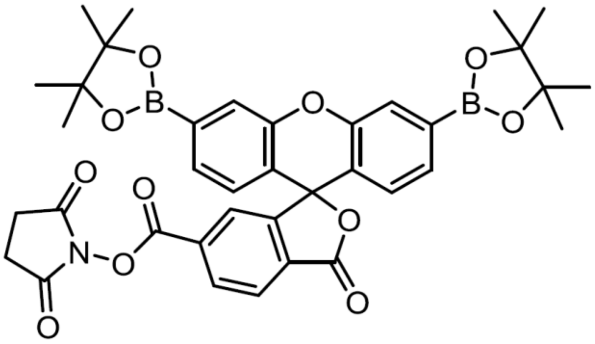

Figure S1. Structure of CPF1 N-hydroxysuccinimide ester (CPF1-NHS).

SNARF2 N-hydroxysuccinimide ester (SNARF2-NHS) (Figure S2) was prepared according to literature [1]. The solid product was characterised by 500 MHz 1HNMR in d6-acetone for comparison to literature: δ(ppm) 8.65 (s, 1H), 8.50 (d, 1H, J = 9.1 Hz), 8.48 (d, 1H, J = 8.1 Hz), 7.59 (d, 1H, J = 7.9 Hz), 7.38 (d, 1H, J = 8.7 Hz), 7.32 (dd, 1H, J1 = 9.0 Hz, J2 = 2.3 Hz), 7.25 (d, 1H, J = 2.2 Hz), 6.84-6.81 (m, 2H), 6.78 (d, 1H, J = 9 Hz), 6.58 (dd, 1H, J1 = 9 Hz, J2 = 2.5 Hz), 3.74 (br. s, 4H), 3.49 (q, 4H, J = 5.3 Hz), 1.21 (t, 6H, J = 4.9 Hz).

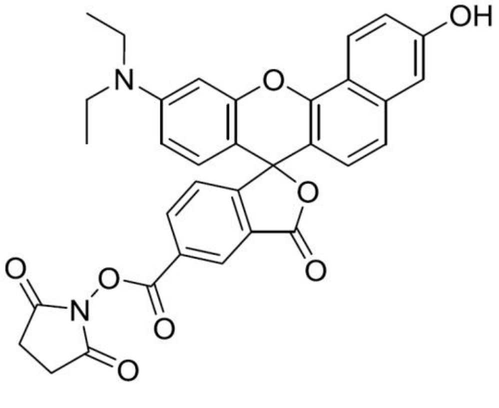

Figure S2. SNARF2 N-hydroxysuccinimide ester (SNARF2-NHS).

2. Fluorescence Spectra of Functionalised Fibre Tips

Figure S3 shows the normalised fluorescent spectra of the fibre probe when excited with either 488 nm or 532 nm. It clearly shows the ability of the probe to distinguish the SNARF signal (532 nm excitation) from the increase in fluorescence from CPF1 (473 nm excitation).

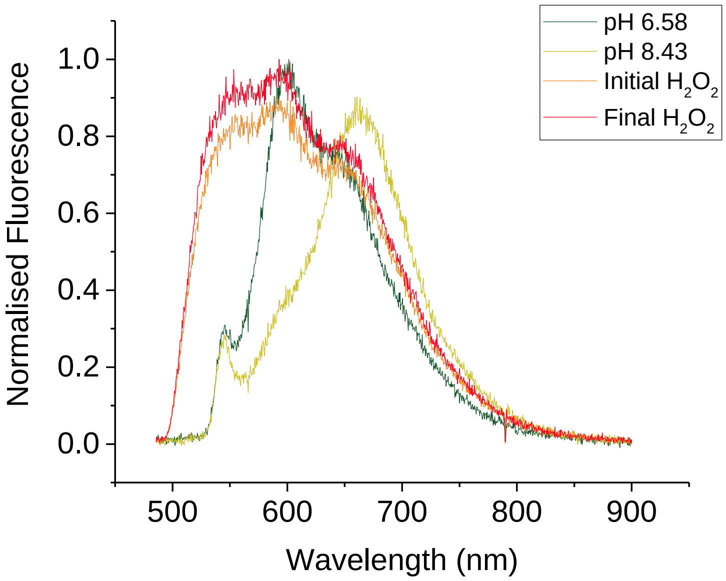

Figure S3. Fluorescence spectra of functionalised optical fibre dipped into various solutions: pH 6.5 and 8.4 in the absence of H_2O_2, excitation 532 nm. Initial (0 min) and final (20 min) when in 100 μM H_2O_2 at pH 7.4, excitation 488 nm. The spectra were normalised at the fluorescent peak of 600 nm for visual comparison.

Figure S4 shows the fluorescent ratio of functionalised probes dipped first into a solution pH 6.5, then into a pH 8.0 buffer. After the basic solution, probes were then inserted into the pH 6.5 solution a second time and finally into the pH 8.0 buffer. The graph exhibits a good correlation between each on both ascending in basicity and descending, suggesting the probes suffer from minimal hysteresis.

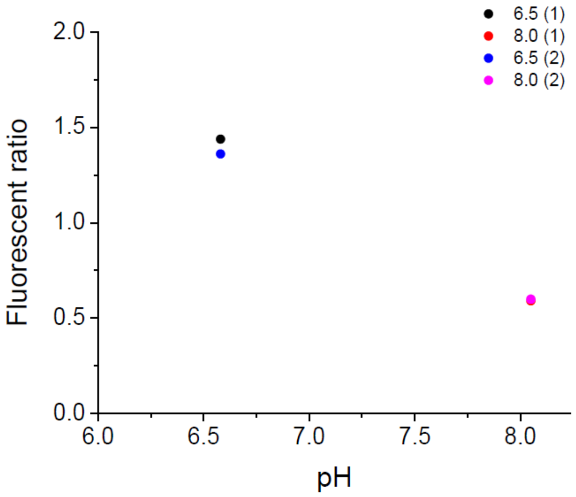

Figure S4. Fluorescent ratio of probes dipped into solutions of varying pH multiple times to determine if any hysteresis is shown.

References

1. Srikun, D.; Albers, A.E.; Chang, C.J. A dendrimer-based platform for simultaneous dual fluorescence imaging of hydrogen peroxide and pH gradients produced in living cells. *Chem. Sci.* **2011**, *2*, 1156–1165.
2. Purdey, M.S.; Connaughton, H.S.; Whiting, S.; Schartner, E.P.; Monro, T.M.; Thompson, J.G.; Aitken, R.J.; Abell, A.D. Boronate probes for the detection of hydrogen peroxide release from human spermatozoa. *Free Radic. Biol. Med.* **2015**, *81*, 69–76.
